# The Influence of Distance and Level of Service Provision on Antenatal Care Use in Rural Zambia

**DOI:** 10.1371/journal.pone.0046475

**Published:** 2012-10-04

**Authors:** Nicholas N. A. Kyei, Oona M. R. Campbell, Sabine Gabrysch

**Affiliations:** 1 Public Health Division, 37 Military Hospital, Accra, Ghana; 2 Faculty of Epidemiology and Population Health, London School of Hygiene and Tropical Medicine, London, United Kingdom; 3 Epidemiology and Biostatistics Unit, Institute of Public Health, University of Heidelberg, Heidelberg, Germany; Institute of Clinical Effectiveness and Health Policy, Argentina

## Abstract

**Background:**

Antenatal care (ANC) presents important opportunities to reach women with crucial interventions. Studies on determinants of ANC use often focus on household and individual factors; few investigate the role of health service factors, partly due to lack of appropriate data. We assessed how distance to facilities and level of service provision at ANC facilities in Zambia influenced the number and timing of ANC visits and the quality of care received.

**Methods and Findings:**

Using the 2005 Zambian national Health Facility Census, we classified ANC facilities according to the level of service provision. In a geographic information system, we linked the facility information to household data from the 2007 DHS to calculate straight-line distances. We performed multivariable multilevel logistic regression on 2405 rural births to investigate the influence of distance to care and of level of provision on three aspects of ANC use: attendance of at least four visits, visit in first trimester and receipt of quality ANC (4+ visits with skilled health worker and 8+ interventions).

We found no effect of distance on timing of ANC or number of visits, and better level of provision at the closest facility was not associated with either earlier ANC attendance or higher number of visits. However, there was a strong influence of both distance to a facility, and level of provision at the closest ANC facility on the quality of ANC received; for each 10 km increase in distance, the odds of women receiving good quality ANC decreased by a quarter, while each increase in the level of provision category of the closest facility was associated with a 54% increase in the odds of receiving good quality ANC.

**Conclusions:**

To improve ANC quality received by mothers, efforts should focus on improving the level of services provided at ANC facilities and their accessibility.

## Introduction

Even though significant progress has been made in reducing maternal deaths globally since the 1990s [1]–[3], many women and their newborns are still dying from preventable pregnancy- and birth-related complications, especially in sub-Saharan Africa [4]. A review of interventions for improved maternal, newborn and child survival in sub-Saharan Africa estimated that nearly 4 million lives could be saved if existing interventions along the continuum of care reached 90% of families [5].

Childbirth is the time when most deaths occur, and thus increased attention has been paid to intrapartum care in recent years. Antenatal care (ANC), while not sufficient to reduce maternal mortality on its own, still presents an important opportunity to reach women with a number of interventions crucial for their health and that of their babies [6]. In the past, organization of ANC in most low-income countries emphasized risk screening for pregnant women during numerous visits, but this approach has been shown to have limited effectiveness [7]. Today, the focused ANC model recommends interventions known to be effective, with the aim of detecting and treating complications during a reduced number of visits [7]. Thus, quality of care at ANC is now emphasized over the quantity of visits.

Zambia has adopted the focused ANC strategy recommended by WHO. The proportion of pregnant women in Zambia who attend ANC at least once with a skilled provider stands impressively high at 94%, while 74% attend at least the recommended four antenatal visits [8], [9]. These figures are tracked in Countdown to 2015 in relation to MDG 5 [10], however, they provide no information about the content and quality of care received and thus paint an incomplete picture. The “effective coverage”, i.e. the proportion of women who receive ANC “with sufficient quality to be effective” [11], [12], is much lower than mere ANC coverage, i.e. the proportion of women using ANC [4]. This “quality gap”, i.e. “the difference between coverage of the basic package and provision of effective and client friendly care” must be closed, both to “provide effective care and to maintain demand for health services” [4].

Studies of the determinants of ANC use focus mostly on individual and household factors. According to a recent systematic review, only one (qualitative) study [13] considered the influence of quality of antenatal services on ANC use, while several quantitative and qualitative studies studied the role of distance to the closest health facility on ANC uptake [14]. A further quantitative study published after the review investigated the influence of quality of care at facilities on ANC use, narrowly defining quality as ANC delivered by a skilled health worker [15]. This is in line with a review of articles employing Aday and Anderson's behavioural model of utilization which concluded that “the context within which utilization occurs – the role of the environment and provider-related factors – has been largely neglected” in studies on health care use [16]. Environmental and provider-related factors in this review included characteristics of the health care delivery system, for example availability of providers, and characteristics of providers, for example their training [16].

One explanation for the scarcity of studies considering the role of the health service environment in determining service use is the lack of information on the level of service provision at health facilities in existing population survey datasets [17]. Merging datasets containing detailed individual-level information with datasets including information on health services is a solution to this problem [16] and has been used in Zambia for studying the influence of distance and level of care on delivery service use [17]. The Zambian setting presents a rare research opportunity where a low-income country with high maternal and perinatal mortality has suitable national health facility data and household data with geographic coordinates.

We used this approach of linking user and provider datasets to quantify the influence of distance and level of service provision at facilities on ANC use and on quality of ANC received in rural Zambia, taking other important individual-, household-, and community-level determinants into account. The specific objectives of this study were to assess (1) how distance to an ANC facility influences number and timing of women's ANC visits; (2) how level of service provision at the ANC facility influences number and timing of women's ANC visits; (3) how distance to care and level of service provision at the closest ANC facility influence the quality of care received by women.

## Methods

### Data sources

The 2007 Zambia Demographic and Health Survey (DHS) was a nationally representative household cluster survey that interviewed 7,146 women aged 15–49 years [8], [18]. It contains information on antenatal care for 4,148 births that occurred in the five years before the survey (counting twin and triplet pregnancies as one birth), of which 2,664 (64%) were to mothers living in rural areas, the focus of our study. This includes data on the number of ANC visits, the timing (by month of pregnancy) of the first visit, and on eleven items of care provided during ANC. Births to mothers who had only moved to their present location after the birth, or were visiting at the place of interview, were excluded because they received ANC at a different location than where they were interviewed and we thus did not have information on distance at time of pregnancy. Excluding these births left a sample of 2,405 rural births for the main analysis.

The 2005 Zambia Health Facility Census (HFC) [19] covered all of Zambia's around 1400 health facilities including public and semi-public facilities (mission, NGO, etc.) of all levels, as well as major private facilities. Of 1391 health facilities with information on ANC availability, 1299 (93%) were recorded as providing ANC. Information collected included number of ANC days per week, current performance of screening tests for anaemia, syphilis, urine protein and urine sugar, availability of tetanus vaccination, provision of folate/iron supplementation, intermittent presumptive treatment (IPT) of malaria, HIV testing and prevention of mother-to-child transmission of HIV, routine discussion of family planning with pregnant women, and ANC outreach services.

### Exposure variables

The exposure variables of interest are distance to the closest facility providing ANC and level of service provision at the closest facility or within 10 km thereof. Straight-line distances in meters from each DHS cluster to the closest ANC facility of a given level of provision were calculated in the GIS platform ArcView 3.2 (ESRI) using the user-written extension “Nearest Neighbor 3.6”.

For our classification of health facilities according to their level of service provision, we were guided by Donabedian's framework on quality of care assessment [20]–[22] distinguishing between the structure of health care, the actual care delivered (process) and the end result of the interaction between an individual and a health care system (outcome), as well as considering other authors who argued that outcome is a consequence of care rather than a component of care and structure is “the conduit through which care is delivered and received” [23]. We thus based our assessment of level of ANC provision at health facilities mainly on process attributes, particularly on the technical aspects of the provider-client interaction during ANC. The only data on structural inputs was availability of medical staff, while availability of drugs and equipment was implicit in the performance of certain functions.

Based on the recommended interventions for pregnancy care [24], initial data analysis and professional judgment, we developed a framework for assessing level of ANC provision, combining availability of five key antenatal functions, five relevant screening tests, skilled health workers according to country definition (doctors, midwives/nurses & clinical officers) [8], and other pertinent services in the continuum of care [25], [26]. Table 1 shows the framework used to classify ANC facilities in Zambia into three broad levels of service provision: optimum service if they fulfill a strict set of criteria, adequate service if they fulfill a more lenient minimum set of criteria, and inadequate service if otherwise.

**Table 1:** Framework for the classification of level of ANC provision in Zambian health facilities.

Category	Criteria	Minimum requirements for optimum level	Minimum requirements for adequate level
**Opening time**	ANC days per week	3+	1
**Staffing**	Skilled health workers registered^1^	3+	1
**Continuity of care**	Delivery service	Y	No requirement
	EmOC or EmOC referral capacity	Y	No requirement
**Screening tests**	Hemoglobin, Syphilis, Urine protein, Urine sugar, Blood group+Rhesus factor	Any 3+ tests (including urine protein)	Any1 test
**ANC functions**	Folate/iron supplement Tetanus vaccine VCT for HIV PMTCT of HIV IPT of malaria	All 5 functions	Any 3 functions

1 Doctor, nurse/midwife, clinical officer.

Y, Yes; N, No; VCT, voluntary counseling and testing; IPT, intermittent preventive treatment; PMTCT, prevention of mother to child transmission.

### Outcome variables

We explored the effect of distance and level of provision on three binary outcome variables obtained from the DHS: (1) attendance of at least the four recommended ANC visits; (2) ANC visit in the first trimester and (3) receipt of “good quality ANC”. Having received good quality ANC was defined as attending at least four ANC visits with a skilled health worker and receiving more than eight of the eleven following antenatal interventions [27]: weight measured, height measured, blood pressure measured, urine sample taken for analysis, blood sample taken for analysis, voluntary counseling and testing for HIV offered, iron supplementation provided, antimalarial drug provided for intermittent preventive treatment for malaria in pregnancy (IPT), birth preparedness plan discussed, treatment provided for intestinal parasites, and tetanus toxoid vaccination. Where information was missing on an item or mothers did not know, we assumed that the mother did not attend four visits, attended the first visit later than in the first trimester or did not receive good quality ANC.

### Statistical analysis

We performed frequency tabulations to describe outcome and exposure variables in the rural DHS subsample and compared the rural with the urban subsample. Crude measures of association of distance and level of provision with the three binary outcome variables were computed, using two-level random effects logistic regression models to account for clustering at the village level. Similarly, crude measures of association for all important potential confounding variables with the outcomes were computed. Variables that showed an association with the outcome (p<0.15 was chosen to also capture weak associations with our given sample size) and were not considered to be on the causal pathway were added to the models containing both distance to the closest ANC facility and the highest ANC quality available at that distance or 10 km thereof, thus evaluating both distance and level of provision simultaneously and adjusting for each other. We used a forward-fitting approach, including mother's education and household wealth as a priori confounders and adding the other variables in the order of the strength of their separate confounding effects. Variables that caused at least a 10% change in the logOR of distance or quality were considered to be confounders and kept in the model. Three models were built, one for each of the three binary outcome variables. All statistical analysis was done using Stata version 11.

## Results

**Figure 1:**
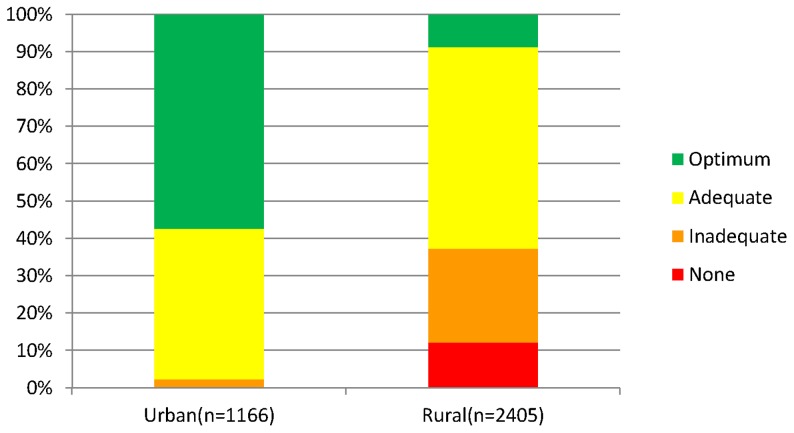
Level of ANC provision within 15 km for urban and rural mothers in Zambia. All urban mothers had access to an ANC facility within 15 km and the majority (57%) had access to an ANC facility providing an optimum level of service within this distance, while 88% of rural mothers lived within 15 km of an ANC facility and only 9% had access to a facility with optimum level of provision within this distance.

All urban mothers had access to an ANC facility within 15 km and the majority (57%) had access to an ANC facility offering an optimum level of service within this distance. In contrast, 88% of rural mothers lived within 15 km of an ANC facility and only 9% had access to a facility with an optimum level of provision within this distance (Figure 1); indicating that the problem of access to a high level of ANC provision is more severe in rural areas. Our analysis of the influence of distance and level of provision on ANC use therefore focused on rural areas.

**Figure 2:**
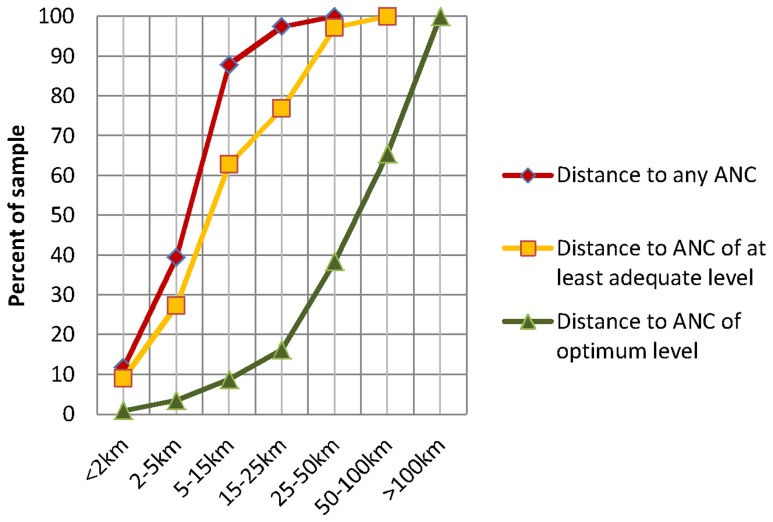
Cumulative distributions of distances to ANC for rural mothers in Zambia. The graph shows cumulative distributions of distances to ANC of various levels of provision for 2405 rural mothers in Zambia. While 40% of rural women lived within 5 km of any ANC facility, distances to facilities with adequate level of provision were farther, and ANC facilities providing optimum services were mostly out of reach for rural women, with 60% living more than 50 km from such a facility.

The cumulative distribution of distances to facilities offering various levels of ANC for rural mothers in Zambia is shown in Figure 2. While 40% of rural women lived within 5 km of any ANC facility, distances to ANC facilities providing an adequate level of service were farther, and ANC facilities with optimum level of provision were mostly out of reach for rural women, with 60% living more than 50 km from such a facility.

While 1461 of 2405 rural mothers (61%) attended the recommended four or more visits, only 505 (21%) also received good quality ANC; i.e. attended four or more visits with a skilled health professional and received eight or more important antenatal interventions. Only 414 rural mothers (17%) attended ANC in the first trimester of pregnancy.

Distance to care did not seem to significantly influence whether a mother had her first antenatal visit in the first trimester or later in either the crude or the adjusted models. By contrast, in the crude model of the effect of level of provision on ANC use in the first trimester, better level of provision at the nearest facility was associated with 26% decreased odds of attending ANC in the first trimester. This surprising association was diminished by adjusting for ethnic group and for women's media use in the cluster and there was only weak evidence for a 23% decrease in odds in the multivariable model. (Table 2)

**Table 2:** Effect of distance and level of provision on various aspects of ANC use in rural Zambia (n = 2405^1^).

OutcomeRisk factors	Crude OR (95% CI)	p-value	Adjusted OR^3^ (95% CI)	p-value
**ANC visit in first trimester**				
Distance (per 10 km increase)	0.85 (0.65–1.10)	0.21	0.88 (0.67–1.16)	0.36
Better level of provision (per category improvement)^2^	0.74 (0.57–0.96)	0.02	0.77 (0.58–1.03)	0.08
**Attendance of at least four ANC visits**				
Distance (per 10 km increase)	0.81 (0.67–0.98)	0.03	0.87 (0.72–1.06)	0.18
Better level of provision (per category improvement)^2^	0.96 (0.79–1.17)	0.69	0.91 (0.75–1.12)	0.39
**Received good quality ANC (i.e. 4+ visits with skilled attendant and 8+ interventions)**				
Distance (per 10 km increase)	0.67 (0.51–0.88)	0.005	0.76 (0.57–1.00)	0.05
Better level of provision (per category improvement)^2^	1.89 (1.44–2.47)	<0.001	1.54 (1.16–2.04)	0.003

1 10, 23 and 23 missing/unknown values for the three outcomes respectively, counted as later than first trimester, less than four visits, and no good quality.

2 Evidence against linearity over categories: p = 0.36, p = 0.74 and p = 0.18, respectively.

3 Confounders included in the adjusted models:

**First trimester visit:** mother's education, household wealth, modern fertility attitude of mother, literacy level of mother and average women's media use in the cluster.

**4+ ANC visits**: mother's education, household wealth, wantedness of pregnancy, modern fertility attitudes of mother and of husband, ethnic group, religion, violence experience, family composition and average men's opinion on women's autonomy in the cluster.

**Good quality ANC**: mother's education, household wealth, ethnic group, modern fertility attitudes of mother, average women's fertility attitudes in the cluster and average women's exposure to health information in the cluster.

In the crude (bivariable) model, we found that the odds of attending the recommended four or more ANC visits reduced by about 20% for each 10 km increase in distance. Adjusting for the mother's individual fertility attitudes, her household wealth, and women's health-care seeking autonomy in the cluster and men's opinion on women's autonomy in the cluster weakened this effect, and there was no evidence that distance decreased the odds of having four or more ANC visits in the multivariable model. There was no evidence that the level of service provision at the closest ANC facility increased the odds of a mother attending the recommended minimum four antenatal visits. (Table 2)

There was evidence for an effect of distance to the closest facility on the quality of ANC received by a mother: In the final model, adjusted for all confounders at individual, household and cluster level, the odds of a woman receiving good quality ANC (at least four visits with a skilled provider and at least eight interventions) decreased by a quarter (24%) for each 10 km increase in distance. Level of service provision at the closest ANC facility (optimum, adequate or inadequate, according to our classification) also strongly influenced whether the mother received good quality ANC (i.e. had at least four visits with a skilled provider and received at least eight interventions). For each category improvement in the level of ANC provision at the closest facility, there was a 54% increase in the odds of receiving good quality ANC in the adjusted model. (Table 2)

## Discussion

Linking data from a national health facility census with data from a national household survey in a geographic information system allowed us to analyze and quantify the relationship between the level of service provision at a facility and women's ANC use, while considering various quality dimensions. We furthermore quantified the influence of distance on ANC use, adjusting for a wide range of confounders, and we studied three different ANC use outcomes (number of visits, timing of first visit, and quality of ANC received).

Our study showed that most rural women in Zambia lived far away from facilities providing an optimum level of service, and that both farther distance to and level of provision at the closest ANC facility were associated with the quality of ANC received by expectant mothers in rural Zambia. Timing of first ANC visit, however, did not seem to be influenced by distance to the closest ANC facility, nor did better level of provision at the closest facility lead to earlier ANC attendance or a greater number of ANC visits.

Our findings on distance to a health facility and ANC use agree with the results of several previous qualitative studies and some quantitative studies [14], in particular with the results of studies in Kenya and rural Haiti that used DHS data with additional community information on health facility access. These both found that an increase in distance to the nearest health facility led to fewer antenatal visits but did not influence the timing of the first antenatal visit [28], [29]. We also found no effect of distance on timing of the first visit. While there was a crude effect of distance on the odds of having four or more visits in our data, we found there was little evidence left for this effect after adjusting for (an unusually large range of) confounders.

The effect of distance on delivery in a health facility in Zambia, using the same datasets, was stronger [17]. This is not surprising, given that ANC can be attended over many months, thus allowing the woman to wait for a transport opportunity to overcome distance, while delivery care needs to be sought at a specific moment in time, sometimes even at night. Attendance in the first trimester of pregnancy, also a long time period, is clearly not determined by geographic access, but likely by other factors, for instance cultural issues around making the pregnancy publicly known can play a role [13].

Studies investigating the influence of distance on the quality of ANC received are scarce, although the information to construct such an outcome variable is available in most DHS datasets. Since focused ANC was introduced, the aim is not only to achieve a sufficient number of visits, but also relevant content during visits [7]. When studying determinants of ANC use, it seems reasonable therefore to use quality of ANC received as an outcome. It is interesting to note that in our study, effect sizes were strongest for this outcome.

The level of service provision, reflecting aspects of quality of care provided at the facility is considered an important determinant of service use [30]. We showed previously that level of emergency obstetric care (measured as availability of signal functions, health workers, electricity, opening hours and referral capacity) influences use of delivery care in Zambia [17]. In this study, we found that the level of antenatal service provision (measured as availability of key functions, screening tests, skilled health workers and opening times) did not seem to affect the number of ANC visits attended, but it did affect the quality of ANC received, as would be expected.

Another quantitative study on quality of care and ANC use found that in Sudan, besides shorter walking time, better quality of care at the facility was associated with a higher proportion of women using ANC monthly from the second trimester onwards [15]. However, that study used the information on whether ANC was provided to the woman by a skilled health worker as a proxy for the quality of care at the facility. This seems methodologically flawed given that women with complications are likely to seek ANC more often and to see more highly skilled providers, thus leading to a spurious association and making the study difficult to interpret.

The overall poor first trimester attendance of ANC in Zambia raises questions about whether women are aware of the importance and expected content of an early first antenatal visit or whether ANC is rather still seen as a check-up before birth to ensure everything is normal. One could speculate that the technical quality of care at ANC facilities may not influence women's early care-seeking behaviour because they don't appreciate the meaning of the ANC interventions, but are rather influenced by interpersonal quality of care. That is to say that the personal experiences a woman had earlier with facility staff, or experiences her friends and family had, may greatly impact on her care-seeking behaviour. Our unexpected finding that a higher level of provision at the closest facility (adjusting for distance) was weakly associated with a later first antenatal visit is difficult to explain. If it is not a chance finding (p-value: 0.08), it could be due to the fact that technically assessed quality of care captured as the level of service provision is not the same as quality of care as perceived by women, and higher-level facilities may offer less personal care – or charge higher costs. A study on ANC use among women in rural Kenya found that lower perceived quality of care was significantly associated with a later first ANC visit [31]. We unfortunately did not have information on perceived quality of care in our datasets.

The interpretation of our findings needs to take into account certain study limitations. The 2005 Zambia Health Facility Census provides only a snap-shot picture of health facility quality at one point in time. The availability of services may have varied during the five-year period (2002–2007) for which the 2007 Zambia DHS recorded ANC use, and the situation today is also likely to be different. On the other hand, using secondary data allowed us to study all of Zambia with a large variety of facilities being considered. This made it possible to overcome previous limitations in investigating the influence of level of service provision, which requires a large range of facilities to be studied. While the DHS covered a large national sample of women, the restriction of ANC information in the DHS on last births and our exclusion of movers left a rural subsample of 2405 births. A larger sample size would have enabled us to draw firmer conclusions.

Non-differential misclassification of distance, due to errors in the geographic coordinates (to which the geoscrambling practice of Measure DHS contributes [32]) and due to differences between straight-line distance and real distance along roads and paths (data on roads and terrain were not available) is likely to have led to an underestimation of the true distance effects. Finally, the Health Facility Census was not designed to specifically assess the level of ANC provision, and thus potentially important information, such as opening hours for ANC per day and number of health workers with ANC-specific skills were not collected, nor did we have information on client satisfaction. We recognize however that quality of care is a multi-dimensional entity and feel our use of proxy measures such as days of ANC provision per week and number of skilled health workers registered in our classification of ANC quality do capture some elements of quality as evidenced by the plausible association seen.

## Conclusions

This study provides evidence that quality of ANC received by expectant mothers in rural Zambia is strongly influenced by the level of services provided at the closest ANC facility and to a lesser degree by distance to care. This suggests that to improve ANC quality received by mothers, efforts and resources should focus on improving the level of service provision at ANC facilities. The need to focus more on quality of care received by expectant mothers has been echoed in a number of recent calls [10], [33]–[35].

This implies scaling-up effective screening tests and interventions towards universal coverage, especially in rural health centers which make up the majority of ANC providers. As most rural mothers live within reasonable distance of an ANC facility providing an adequate level of service (Figure 2), it may be useful to start by upgrading and improving the level of provision at these facilities to transform them from just adequate to optimum facilities capable of performing all essential ANC screening tests and interventions. In fact, there have been a number of improvements in recent years. The Zambian Ministry of Health, together with its partners, has bought necessary equipment and improved recruitment and placement of midwives [36]–[38].

Further research may be needed to better understand the factors influencing ANC care-seeking in the first trimester of pregnancy, as well as the difference between perceived quality of care and technically assessed quality of care at a facility, as this could help understand client behavior and form the basis for future behavior change campaigns. As antenatal care-seeking overall is rather good, the “quality gap” for ANC is much larger than the “coverage gap” [4], and thus the responsibility for improvement lies foremost with the health sector. It would be helpful to conduct implementation research while improving quality of care at health facilities to gather operational knowledge on how this is best done, and thus help to close the “quality gap”. It would furthermore be important to study the impact of improved access and level of service provision on the mortality and morbidity of mothers and babies in Sub-Saharan Africa.
